# Long-term safety of human retinal progenitor cell transplantation in retinitis pigmentosa patients

**DOI:** 10.1186/s13287-017-0661-8

**Published:** 2017-09-29

**Authors:** Yong Liu, Shao Jun Chen, Shi Ying Li, Ling Hui Qu, Xiao Hong Meng, Yi Wang, Hai Wei Xu, Zhi Qing Liang, Zheng Qin Yin

**Affiliations:** 1 Key Laboratory of Visual Damage, Regeneration and Repair, Southwest Eye Hospital, Third Military Medical University, Chongqing, 400038 China; 2 Department of Gynecology and Obstetrics, Southwest Hospital, Third Military Medical University, Chongqing, 400038 China

**Keywords:** Progenitor cell, Visual improvement, Retinitis pigmentosa, Cell transplantation, Retina

## Abstract

**Background:**

Retinitis pigmentosa is a common genetic disease that causes retinal degeneration and blindness for which there is currently no curable treatment available. Vision preservation was observed in retinitis pigmentosa animal models after retinal stem cell transplantation. However, long-term safety studies and visual assessment have not been thoroughly tested in retinitis pigmentosa patients.

**Methods:**

In our pre-clinical study, purified human fetal-derived retinal progenitor cells (RPCs) were transplanted into the diseased retina of Royal College of Surgeons (RCS) rats, a model of retinal degeneration. Based on these results, we conducted a phase I clinical trial to establish the safety and tolerability of transplantation of RPCs in eight patients with advanced retinitis pigmentosa. Patients were studied for 24 months.

**Results:**

After RPC transplantation in RCS rats, we observed moderate recovery of vision and maintenance of the outer nuclear layer thickness. Most importantly, we did not find tumor formation or immune rejection. In the retinis pigmentosa patients given RPC injections, we also did not observe immunological rejection or tumorigenesis when immunosuppressive agents were not administered. We observed a significant improvement in visual acuity (*P* < 0.05) in five patients and an increase in retinal sensitivity of pupillary responses in three of the eight patients between 2 and 6 months after the transplant, but this improvement did not appear by 12 months.

**Conclusion:**

Our study for the first time confirmed the long-term safety and feasibility of vision repair by stem cell therapy in patients blinded by retinitis pigmentosa.

**Trial registration:**

WHO Trial Registration, ChiCTR-TNRC-08000193. Retrospectively registered on 5 December 2008.

**Electronic supplementary material:**

The online version of this article (doi:10.1186/s13287-017-0661-8) contains supplementary material, which is available to authorized users.

## Background

Retinitis pigmentosa is an inherited retinal dystrophy that is characterized by the onset of night blindness, the early loss of peripheral vision, and later the loss of central vision [1]. Retinitis pigmentosa is related to different genetic etiologies, all of which induce the death of photoreceptors. Therefore, the identification of causative genes must be a prerequisite if gene therapies are to be applied to treat retinitis pigmentosa patients [2–4]. Compared to gene therapies, cell transplantation might have good potential to rescue or replace dysfunctional photoreceptors. Schwartz et al. reported on mid-term and long-term outcomes when using human embryonic stem cell (hESC)-derived retinal pigment epithelial (RPE) cells to treat dry atrophic age-related macular degeneration and Stargardt's macular dystrophy [5, 6]. Their studies provided the first evidence that hESC-derived cells can be used as a new form of therapy to treat retinal degeneration.

Studies using retinitis pigmentosa animal models indicated that the transplanted cells were able to differentiate into photoreceptors, integrate into the host retinas, and rescue vision [7–11]. These findings suggested that it is possible to transplant human RPCs to treat retinitis pigmentosa patients. Currently, retinal progenitor cells (RPCs) are usually derived from fetal retinas, embryonic stem cells (ESCs), and induced pluripotent stem cells (iPSCs). With regard to the source of RPCs, fetal-derived RPCs may have an advantage for cell therapy. Firstly, RPCs derived from fetal neural retinas have low immunogenicity and can stably maintain their characteristics over many passages [12–14]. RPCs derived from ESCs or iPSCs require a longer time and more steps to induce in vitro. Secondly, transplanting ESCs or iPSCs carries the potential risk of tumor formation and gene mutation [15, 16].

The most relevant previous clinical studies to treat retinal degeneration used fetal retinal sheets and immature neural retinal cells, and there were no obvious signs of immune rejection; however, parallel animal studies suggested there was only limited progenitor cell integration into the neural retina [17–19]. Subsequent reports suggested that only transplanted cells isolated from fetuses at a specific gestational stage and already committed to a photoreceptor cell fate could facilitate visual recovery [7–9]. This means that the transplanted cells should be photoreceptor precursors before being delivered to patients. Candidate cells for this treatment exclusively express the cone-rod homeobox-containing gene (CRX), but no expression of Opsin (the main chromophore of the mature photoreceptor) [20]. Human fetal neural retinal cells from second-trimester fetuses can be expanded into a large number of undifferentiated cells in vitro and mature retinal cells [14]. These cells can be ideal sources for transplantation when considering a dose-response relationship in pre-clinical and clinical studies.

We previously reported on the technical feasibility of human retinal transplantation using a mini-pig model and a pars plana vitrectomy approach [21]. In this study, we describe the preparation and isolation of highly enriched human RPCs derived from early developmental fetuses and investigated the potential of these cells to integrate into the host retinas of RCS rats and to restore a visual response. We then initiated a clinical trial in patients with retinitis pigmentosa to investigate the safety, immunological response, and efficacy of human fetal-derived RPC subretinal transplantation using pars plana vitrectomy approach.

## Methods

### Study design and ethics

The animal study was approved by the Laboratory Animal Welfare and Ethics Committee of the Third Military Medical University, and the clinical trial was approved by the Medical Ethics Committee of Southwest Hospital, the Third Military Medical University. We conducted a phase I clinical trial assessing the safety of RPC transplantation into RP patients over a 24-month follow-up period. This trial was conducted in the Southwest Hospital, China. Recruitment of patients started in May 2008 and the study was completed in September 2013. The research adhered to the principles of the Declaration of Helsinki, and written informed consent and surgical consent were obtained from all patients (WHO Trial Registration, ChiCTR-TNRC-08000193).

### RPC isolation

The culture of RPCs was performed under Good Manufacturing Practice (GMP) conditions in the Cell Biology Therapy Center, Southwest Hospital, Third Military Medical University. This center has been awarded GMP certification and qualified for the production of RPCs.

Ocular tissues of 12- to 16-week-old aborted fetuses were collected from the embryonic tissue bank of the Department of Obstetrics, Southwest Hospital, according to the good tissue practice guidelines. Donors provided informed consent and were not compensated for the use of their terminated fetal tissue for research. Fetal neural retinas were cut into pieces, rinsed, digested at 37 °C for 20–30 min with Tryple (CTS, Gibco) and diluted by the addition of 3 ml medium (Ultraculture; Lonza)). The tissue was dissociated by gentle agitation for 10 s and the suspension was settled for 2 min. The supernatant containing the precursors was carefully decanted into a new tube with fresh medium, and the remaining pellet was discarded. The collected supernatant was centrifuged for 5 min at 2400 g and re-suspended in Ultraculture supplemented with 10 ng/ml human epithelial growth factor (EGF; Peprotech) and 20 ng/ml human basic fibroblast growth factor (bFGF; Peprotech) [14]. Cells were plated onto matrigel-coated tissue culture surfaces (Cellstart CTS, Invitrogen) and placed in an incubator for 120 min. The majority of non-neuronal cells adhered to the bottom of the plate, whereas neuronal progenitor cells remained in suspension such that the non-adherent suspension can be collected for primary culture and expansion [22]. Freshly purified RPCs were supplemented with Ultraculture, B27, N-2, 20 ng/ml human EGF, and 20 ng/ml human bFGF, placed on fibronectin-coated (10 μg/ml) plates and placed in an incubator (37 °C, 5% CO_2_). RPCs at passage three were used for the following study. The viability of RPCs was determined using Trypan blue staining (0.4%; GIBCO).

### Immunocytochemistry

The RPCs were plated onto glass cover slips. Primary antibodies were used to characterize the cells (PAX6, 1:100, Santa Cruz; CRX, 1:50, Santa Cruz; Nestin, 1:500, BD Bioscience; Sox2, 1:1000, Chemicon; GFAP, 1:1000, Chemicon). Cy3 (1:1000, Santa Cruz) was used as the secondary antibody and the slides subsequently imaged using a confocal microscope (Leica TCS NT, Leica Microsystems).

### Flow cytometry

In brief, RPCs were prepared in a cell suspension, incubated with the primary antibodies (1:30 for Nestin, PAX6, SOX2, and GFAP) or isotype control (1:30; BioLegend), washed, and then incubated with fluorophore-conjugated secondary antibodies (1:30). Cells were analyzed on a fluorescence-activated cell sorting Calibur system (FACS, BD Biosciences, San Jose, CA, USA). The ratio of positive cells within the gated population was estimated based on comparison with species-specific isotype control. Ten thousand events/sample were collected, stored for analysis, and the experiments were repeated three times.

### Real-time quantitative polymerase chain reaction (RT-qPCR) analysis

Total RNA was extracted from the RPCs by the RNeasy Mini Kit (Qiagen) and cDNA generated using the iScript cDNA Synthesis Kit (Bio-Rad) according to the manufacturer’s instructions. RT-qPCR) was carried out using a Power SYBR Green PCR Master Mix on the 7500 Real-time PCR System (Applied Biosystems). hESC line H1, which was a gift from Shanghai Institute of Biochemistry and Cell Biology, was used for comparison with RPCs. RT-qPCR assayed for the hESC markers Nanog and OCT4, and for the RPCs markers PAX-6, Six6, Crx, and recoverin. Relative gene expression was assayed in triplicate replicates normalized to the GAPDH signal present in each sample. The expression levels of cell markers detected in RPCs were normalized to that of an hESC sample which served as the zero set point.

### Differentiation of RPCs into photoreceptors

Retinoic acid (10 μM; Sigma) was added into serum-free conditioned medium and the cells were cultured for 2 weeks in order to induce the RPCs to differentiate into mature photoreceptors [23]. Cells were then identified by the specific markers recoverin (1:1000, Chemicon) and rhodopsin (1:250, Chemicon). The cellular proliferating properties were examined by anti-Ki67and Ki67 (1:200, Abcam). Cy3-conjugated IgG was used as a secondary antibody.

### Cell transplantation into RCS rats

RPCs were pre-labeled with the fluorescent marker CM-DiI (2 mg/ml; Invitrogen) prior to transplantation. For the efficacy study, RCS rats at 30 days old received an injection with RPCs (*n* = 12); 0.01 M phosphate-buffered saline (PBS) injections were used as a vehicle control (*n* = 6). The right eyes served as the treatment eyes, whereas the left eyes were untreated.

Rats were anesthetized by an intraperitoneal injection of a solution of ketamine (120 mg/kg) and xylazine (20 mg/kg). A scleral hole was created using a 30G needle allowing access to the space between the neural retinal layer and the retinal pigment epithelium layer. A glass micropipette carrying 5 μl of a RPC suspension (1 × 10^5^ cells) was inserted tangentially into the space beneath the degenerating photoreceptor layer at the superior retinal hemisphere. Fundus examination was performed immediately after surgery, and a successful injection was confirmed by a small subretinal fluid bleb. Cyclosporine A (200 mg/L) was given orally from day 1 until sacrifice.

### Functional test after cell transplantation

Electroretinography (ERG) analysis was used to evaluate the improvement in retinal function after cell injections.

Three and six weeks following transplantation, animals were dark adapted for at least 12 h before the ERG test. Anesthesia was performed as above. Pupils were dilated using 1% tropicamide. The active gold lens electrode was placed on each cornea, and the reference and ground electrodes was respectively placed subcutaneously in the mid-frontal area of the head and the base of the tail. Light stimulation was delivered at –5 dB for the dark-adapted test, and all recordings were processed by software supplied by the manufacturer (Diagnosys LLC, MA). The amplitudes of a-waves were measured from the baseline to the cornea-negative peaks, and the amplitudes of b-waves were measured from the cornea-negative peak to the major cornea-positive peak.

### Immunohistology

Rats were sacrificed at 6 weeks post-transplantation. The eyes were fixed in paraformaldehyde in PBS, infiltrated with sucrose, and then sectioned using a cryostat. The injected cells were preliminary identified by the fluorescent marker CM-DiI with fluorescence microscopy. Sections were washed in PBS three times to remove CM-DiI. Mouse anti-human mitochondria (1:200, Abcam) and rabbit anti-human recoverin (1:1000) or rhodopsin (1:250, Chemicon) were used as primary antibodies to detect the transplanted RPCs, and then sections were incubated in the secondary antibodies, Cy3-conjugated AffiniPure goat anti-mouse IgG (1:300) and FITC-conjugated AffiniPure goat anti-rabbit IgG (1:300).

We chose three rats to quantify the percentage recoverin/rhodopsin-positive cells among RPCs. From each rat, three random sections that containing the typical transplant areas were selected. The ratio of double-stained cells among the human mitochondrial-positive cells was considered as the photoreceptor cells differentiated from the grafted RPCs.

To compare the degree of outer nuclear layer (ONL) preservation between RPCs and vehicle groups, the thickness of the ONL was measured on the areas extending 100 μm either side of the injection site.

RPCs were also assessed for tumor formation in the retina. RPCs were injected (as above) into the space beneath the degenerating photoreceptor layer of P30 RCS rats (*n* = 36) and then examined 6 weeks post-transplantation. Hematoxylin-eosin staining was used to examine tumor formation in the injection area.

### Patients

We enrolled eight patients diagnosed with rod-cone dystrophy on the basis of eye examinations, visual field testing, standard full-field fundus fluorescein angiography (FA), and flash (f)ERG according to the standards set by the International Society for Clinical Electrophysiology of Vision (ISCEV) [24]. Patients met the following inclusion criteria: (1) between 18 and 50 years of age; (2) best corrected visual acuity (BCVA) ≤ 20/400 in the operated eye, or a visual field of less than 20°, as assessed by Octopus 101 perimeter; and (3) the vision in the non-operated eye had to be better than the operated eye. Exclusion criteria included evidence of other eye disease such as a cataract that could compromise the interpretation of visual results; the inability to return for follow-up according to pre-planned schedule during the study; and history of intraocular surgery.

### Surgical procedure for cell transplantation into retinitis pigmentosa patients

A standard three-port vitrectomy was performed and the vitreous body was removed from the inner limiting membrane of the retina. Using a 39G retinal hydrodissection cannula (Storz, USA), a minimally invasive retinotomy was performed temporally or superatemporally to the macula and near the arcade vessels. A RPC suspension (100 μl containing ~ 1 × 10^6^ cells) was slowly injected into the presumptive space thus creating a small retinal bleb (Additional file 1: Video S1). Cells were assessed prior to transplantation for microbial contaminants and endotoxin. Post-surgical treatment followed standard procedures for patients receiving three-port vitrectomy.

### Clinical evaluation

Seven patients were followed for 24 months and one patient for 12 months. BCVA was measured three times at each visit using the Early Treatment Diabetic Retinopathy Study (ETDRS) chart. Data were then converted into logMAR (log of the minimum angle of resolution) scores according to the formula 1.1 + log_10_ (designed distance/testing distance) – 0.02 × number of letters [2]. A high logMAR score indicates poor vision. Patients with only hand-motion vision were assigned a score that was one line lower than the largest printed line on the 4-m chart (<20/1600).

On each follow-up visit (1, 2, 3, 6, 9, 12, and 24 months post-transplantation), photographs and autofluorescence/fluorescein angiography of the fundus were performed using a Heidelberg HRA II system (Heidelberg Engineering GmbH, Germany). High-resolution optical coherence tomography (OCT; OCT-1000 System, Topcon) and Spectral Domain OCT (SD-OCT, Spectralis 3 Mode OCT, Heidelberg Engineering) were used to evaluate retinal structure. Bilateral full-field ERGs were recorded using a Roland electrophysiology system (RETIscan, Roland Consult, Germany) with ERG-jet contact lens electrodes. For the ERG analyses, the pupils were dilated with 1% tropicamide and the patient dark-adapted for 30 min. ISCEV standard dark-adapted and light-adapted ERGs were recorded.

Pupillary light reflexes in the dark-adapted (40 min) state were evaluated using a custom-built computerized pupillometer and a modified commercial spherical Ganzfeld according to the method of Aleman et al. [25]. Five continuous 200-ms blue stimuli with intensities ranging from –2.8 to 0.85 log scot-cd/m^2^ and six white stimuli ranging from –1.5 to 2 log scot-cd/m^2^ were applied to elicit a transient light reflex; each stimulus (from low to high) was followed by a 15-s dark recovery period [26]. It was difficult to analyze the amplitude changes for individual pupillary reflexes due to large amplitude variations elicited by the same stimulus. A response criterion of 0.3 mm was used to define a response threshold. The threshold values were converted to ranked data. A one-level improvement corresponded to a one-level decrease in the threshold.

### Statistical analysis

Data are given as the mean ± SD. Comparisons were made using a two-tailed paired *t* test for visual acuity. The treatment effect was compared to the baseline condition and that in the follow-up time points. Differences in BCVA (logMAR) were obtained for each patient at 0, 1, 2, 3, 6, 9, 12, and 24 months post-transplantation. The differences following treatment at each time point were normalized to the baseline measurement obtained at month 0 in order to perform comparisons across patients. A chi-square test was used to assess differences in the pupillary light reflexes between the operated and non-operated eye. All statistical tests were considered significant if *P* ≤ 0.05.

## Results

### Human fetal-derived RPC transplants in RCS rats

**Fig. 1:**
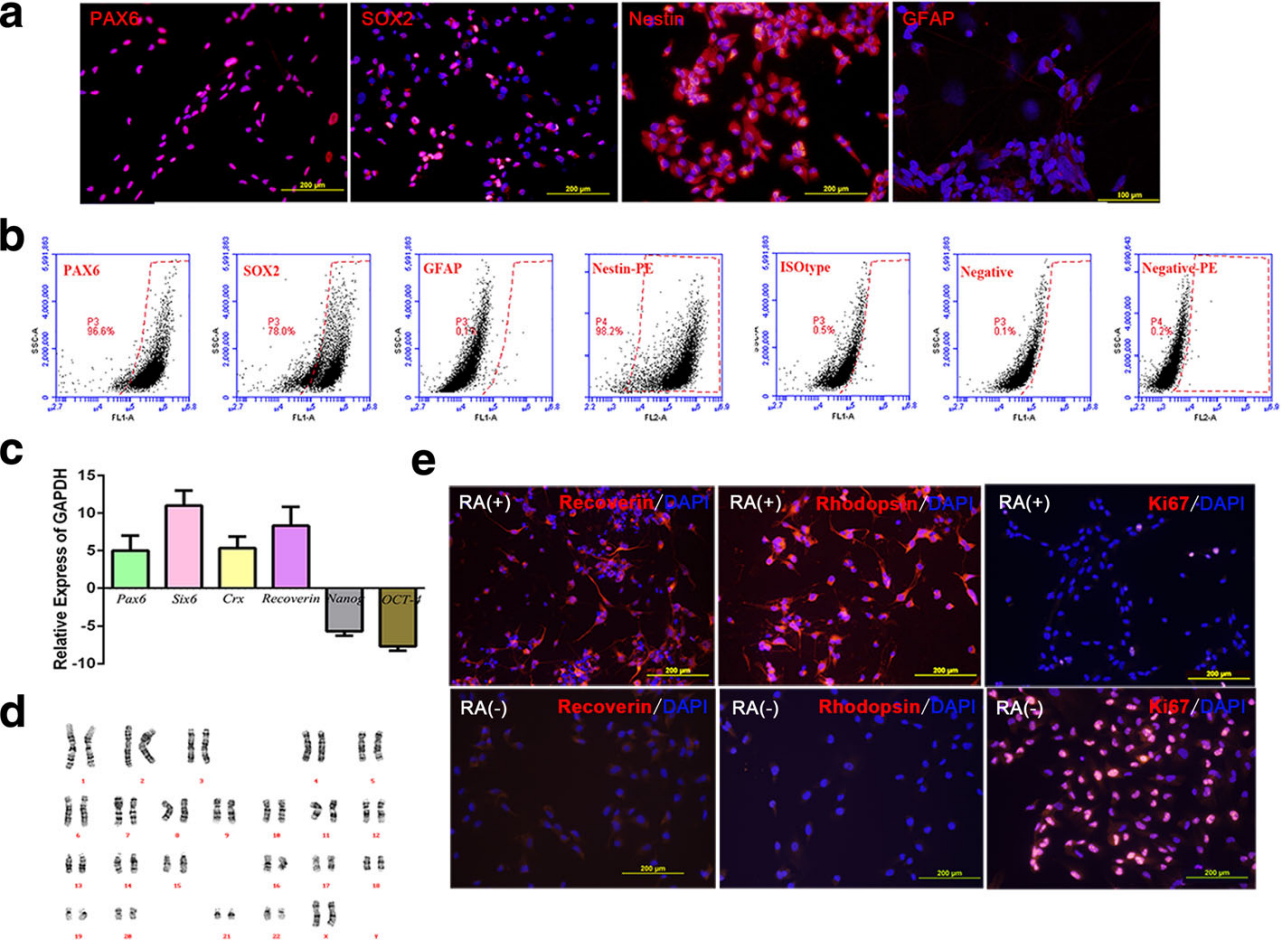
Characterization of retinal progenitor cells (RPCs). **a** Undifferentiated RPCs were stained with early eye field markers, including paired box protein 6 (*PAX6*) and SRY (sex determining region Y)-box 2 (*SOX2*), and for the immature neural cell marker Nestin. RPCs were stained negative for glial fibrillary acidic protein (*GFAP*). **b** Flow cytometry profiles of RPCs for subpopulations expressing PAX6, SOX2, Nestin, and GFAP, respectively. **c** The graph shows gene expression of retinal progenitor and mature retinal markers (fold increase) in hRPCs compared with human embryonic stem cells (hESCs) assayed by real-time quantitative polymerase chain reaction (RT-qPCR). **d** Normal female (46 XX) karyotype of the clinical RPCs. **e** Photoreceptor differentiation by retinoic acid (*RA*) treatment in vitro. RA(+) groups showed RPCs were positive for the mature photoreceptor markers recoverin and rhodopsin and negative for Ki67 after RA inducing. RA(-) groups are the controls

RPCs after three passages were characterized by checking the expression of Nestin (98%), Pax6 (96.6%), and Sox2 (78%) using immunocytochemistry or flow cytometry (Fig. 1a and b). There were limited glial cells in the population of PRCs (0.1% GFAP^+^ cells). Gene expression analysis confirmed that the typical early eyecup transcription factor genes Pax6 and Six6 (Fig. 1c) increased 5–10 fold compared to that seen in hESC cultures; the expression of photoreceptor precursor markers, such as recoverin and CRX, was much higher in the RPCs. In contrast, the expression of the pluripotency markers NANOG and OCT4 clearly decreased by 5–10 times compared to hESCs. RPCs were also able to differentiate and express photoreceptor phenotypes (recoverin and rhodopsin) in vitro following treatment with retinoic acid, and lost proliferative properties without Ki67 staining (Fig. 1e). Since RPCs would be transplanted into degenerated retina in retinitis pigmentosa patients, these cells were extensively tested for animal and human pathogens. The final RPC product had normal female (46 XX) karyotype, confirming that the cells were free of microbial contaminants (Fig. 1d).

**Fig. 2:**
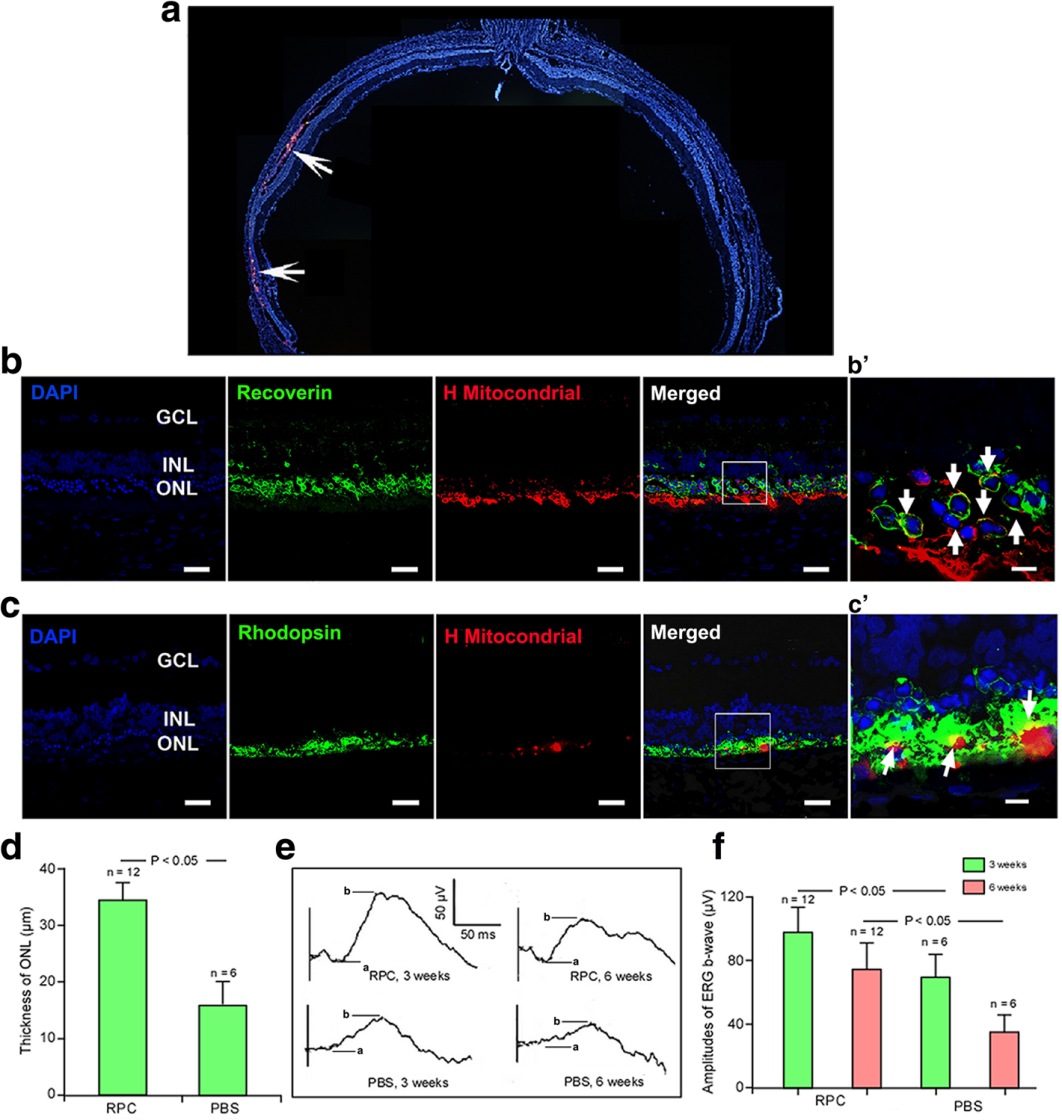
Transplantation of retinal progenitor cells (*RPCs*) repopulated the RCS rat outer nuclear layer and increased electroretinal function. **a** Distribution of the transplanted cells (*arrows*) in the subretinal space at 6 weeks after xenotransplantation indicated by DiI staining. Horizontal cellular migration could be visualized. **b** RPCs stained with anti-human mitochondria were seen in the outer nuclear layer (*ONL*), some cells were double-labeled recoverin. In addition, the ganglion cell layer (*GCL*) and inner nuclear layer (*INL*) were also marked in the host retina. **b’** Enlarged area reflecting the differentiation of transplanted cells. **c,c’** RPCs were double-labeled with anti-human mitochondria and rhodopsin. **d** Mean and standard deviation measurements of the ONL thickness in the RPC-grafted area were significantly higher compared to the control group (*P* < 0.05). **e** Representative electroretinography (*ERG*) (5 dB flash under scotopic conditions) recorded at 3 and 6 weeks after cell transplantation. **f** Mean b-wave amplitude peaks were significantly higher in transplanted animals compared to phosphate-buffered saline (*PBS*) controls at 3 and 6 weeks after cell transplantation (*P* < 0.05). *Scale bars* = 50 μm (**b,c**), 10 μm (**b’,c’**)

Six week following human RPCs transplantation, DiI-labeled cells could be readily identified within RCS retinal sections and, in Fig. 2a, it is clear that transplanted cells had spread across a broad area of the region previously occupied by the photoreceptors. Moreover, immunofluorescence staining confirmed that these cells exclusively expressed human mitochondria, a specific marker of the human species. Most RPCs integrated in the host ONL. Some cells co-expressed recoverin or rhodopsin (Fig. 2b and c). We observed approximately 20.7 ± 3.1% recoverin-labeled and 9.7 ± 1.5% rhodopsin-labeled RPCs in the recipient ONL in each randomized section, indicating the expression of a photoreceptor phenotype and possible photoreceptor differentiation.

Statistical analysis of ONL thickness indicated that transplants were associated with a significantly thicker ONL compared with that in the PBS group (37.2 ± 2.8 μm vs 18.4 ± 2.0 μm, *P* < 0.05; Fig. 2d). Analysis of the recorded ERGs at 3 and 6 weeks post-transplant indicated that the b-wave amplitudes were higher compared to those of the PBS group (*P* < 0.05; Fig. 2e and f). These findings showed that retinal function was profoundly improved after RPC transplantation, which corresponds to the morphological results by ONL thickness measurements.

We then tested the risk of tumor formation by injecting RPCs into the degenerating retinas. Cellular survival was observed in 32 eyes from 36 RCS rats; tumors were not observed in any retinal sections, suggesting the safety of human RPC transplantation in retinitis pigmentosa patients.

### Clinical study of fetal-derived RPC transplantation in retinitis pigmentosa patients

**Fig. 3:**
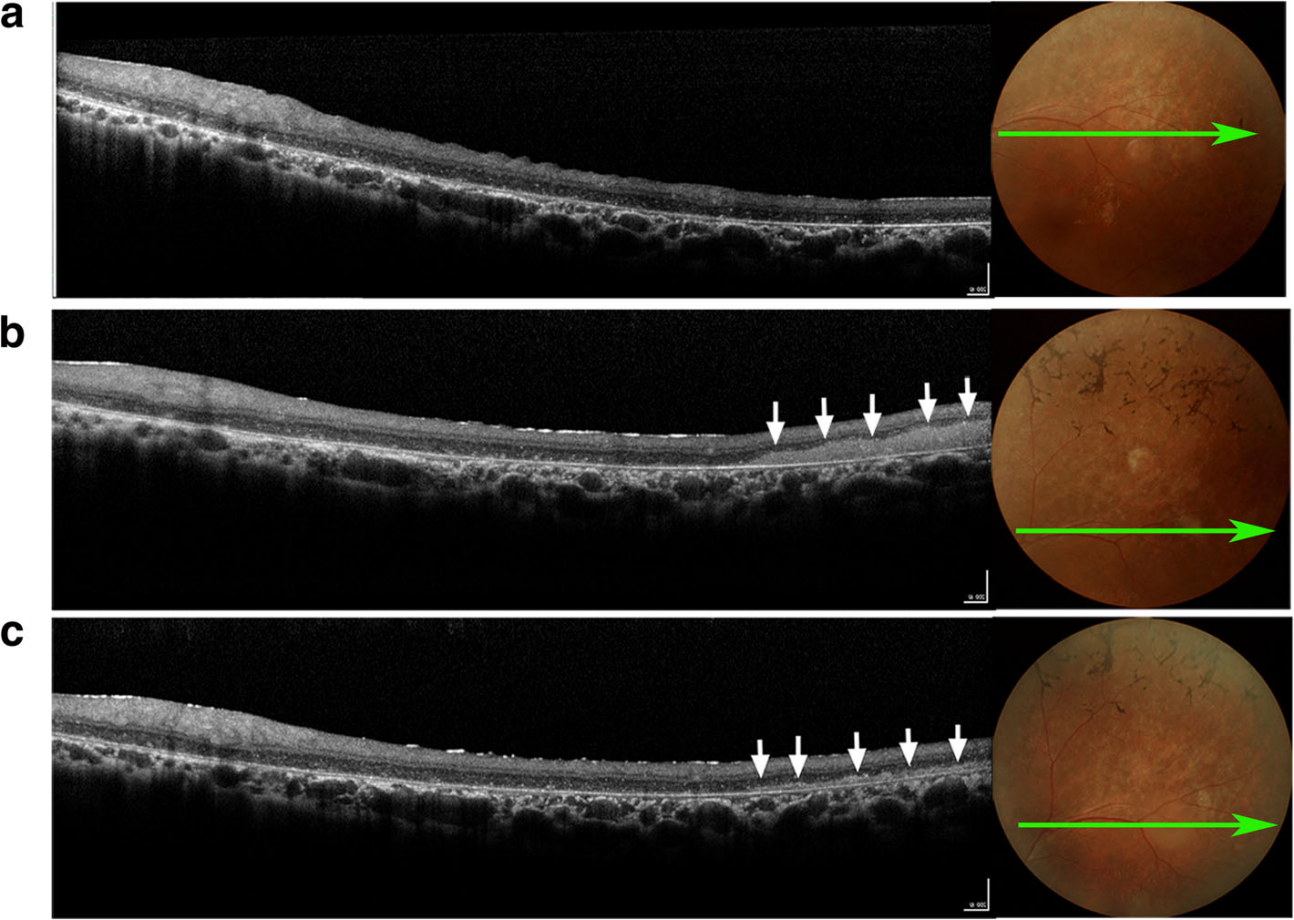
Representative retinal appearance and morphologic features before and after cell transplantation for patient 6. Retinal structures were imaged by optical coherence tomography (OCT) before and after surgery. **a** OCT imaging with horizontal scanning along the superior temporal macular area before surgery. Notice the characteristic of local photoreceptor atrophy. **b** On postoperative day 7, a mass of transplanted cells (*arrows*) is most evident as dense medium reflectivity, which is located between the degenerated photoreceptor layer and retinal pigment epithelium layer. **c** Transplanted cells are still present although the thickness has been noticeably reduced (*arrows*) 1 month after surgery

**Fig. 4:**
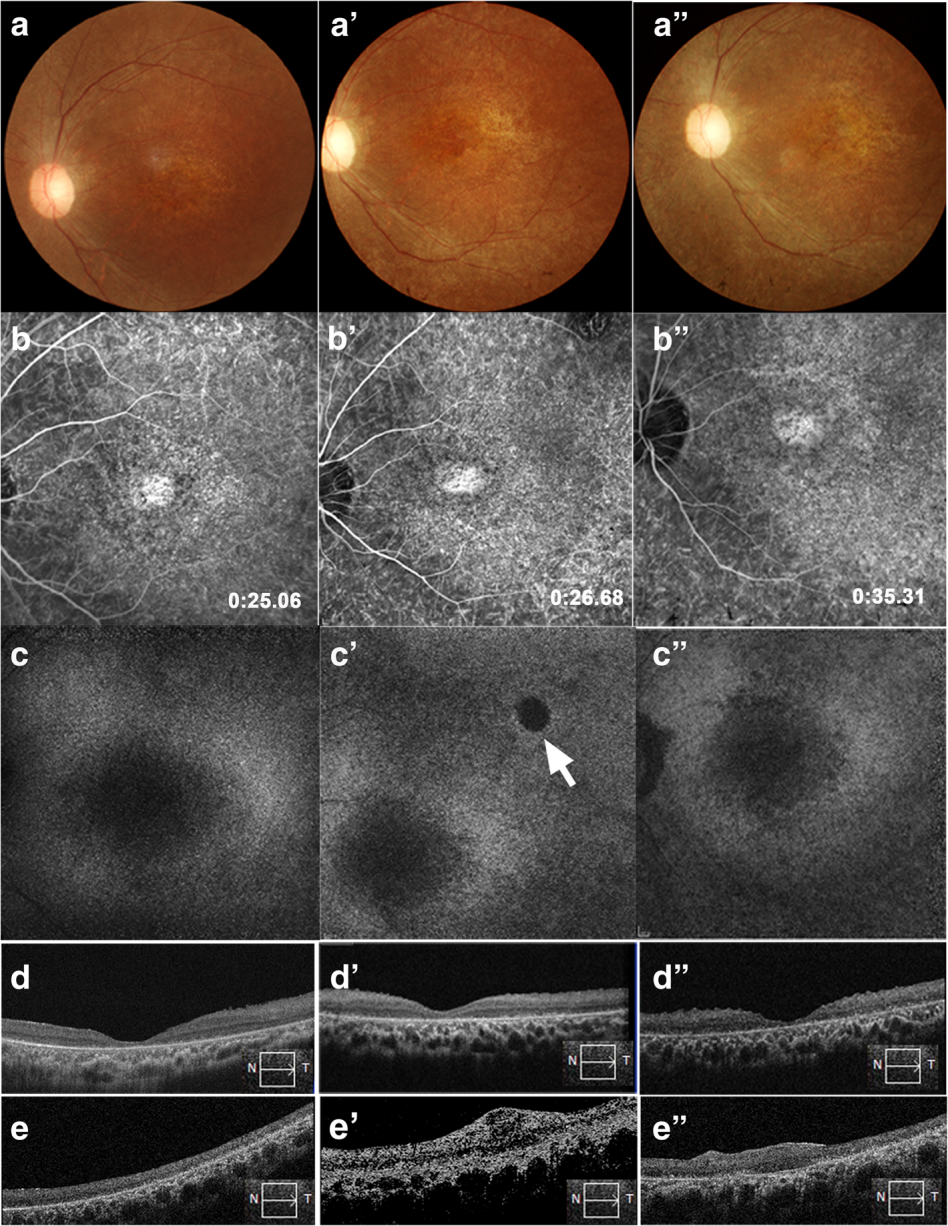
Retinal morphological changes after RPC transplantation in patient 6. **a–d** Baseline images, **a’–d’** 12-month follow-up, and **a”–d”** 24-month follow-up. **a** Color fundus photographs, **b** fluorescein angiograms, **c** autofluorescence imaging in the macular area, **d** foveal optical coherence tomography (OCT), and **e** horizontal OCT scanning along the injection site. **a’,a”** No retinal hemorrhage or edema occurred after RPC transplantation. **b’,b”** The characteristics of fluorescent leakage did not change after transplantation. **c’,c”** No obvious autofluorescence destruction in the macular area after RPC transplantation, except for a minimal area of hypo-autofluorescence (*arrow*). This disrupted RPE layer corresponded to the injection site. **d’,d”** Foveal depression was maintained pre- and postoperatively, indicating that no macular edema occurred. **e** The injection site before surgery (*box* and *arrow* indicate the direction of OCT scanning). **e’,e”** Signs of the injected cells could not be observed at 12 and 24 months post-implant and, in this patient, retinal scarring was evident with local retinal thickening

Preoperatively, the visual acuity of the prospective transplanted eye was 1.37 ± 0.34 logMAR in the eight patients and is equivalent to ~ 20/500 on a Snellen’s vision chart. Vitrectomy and subretinal transplantation proceeded without complications, such as iatrogenic retinal tears, cataracts, or endophthalmitis (Table 1 and Additional file 9: Video S1). All postoperative retinal examinations on day 1 were unremarkable, apart from the formation of the retinal bleb containing the transplanted cells. Clinical examination on day 7 revealed that retinal detachments had not occurred and the OCT analysis clearly showed a thick region of transplanted cells beneath the neural retinal layer (a typical example is shown in Fig. 3b). Although reduced in thickness, the layer of RPCs was still clearly present in OCT scanning 1 month after surgery (Fig. 3c). The reduced thickness at the transplant site suggested that the grafted cells may have migrated within the subretinal space or integrated into other layers of the host retina, in a similar fashion to that seen within the RCS rat retinas. One and two years post-transplantation, it was no longer possible to unequivocally define the region of injected cells using the OCT methodology (Fig. 4 and Additional files 2–7: Figures S2–S7). In some cases the region of the injection site could be defined by the presence of small retinal scarring, characterized by locally thickened retina with OCT scanning. Autofluorescence provided useful information on conditions where the health of the RPE played a key role; areas of hypo-autofluorescence indicated missing or dead RPE cells [27]. Autofluorescence imaging showed a limited hypo-autofluorescence area beneath the corresponding retinal injection site, a sign of local RPE disruption (Fig. 4c’). The restricted area of these regions indicated that our surgery was safe and minimally invasive.

**Table 1:** Summary data for retinitis pigmentosa patients and the retinal progenitor cells

			BCVA (logMAR)		Donor cells	
Patients	Age (years)	Sex	OS	OD	Gestational age (weeks)	Viability
1	42	M	1.56	1.6	14	>98%
2	33	F	1.30	1.3	15	>98%
3	34	M	1.00	0.8	12	90–93%
4	19	M	0.35	0.3	12	95%
5	46	F	1.52	0.9	15	96%
6	19	F	1.30	0.4	12	94%
7	53	F	2.00	1.8	12	95%
8	38	F	0.92	1.0	12	93%

*BCVA* best corrected visual acuity, *F* female, *M*, male, *MAR* minimum angle of resolution, *OD* right eye without treatment, *OS* left eye with transplant

Fluorescein angiography indicated that our procedure did not lead to changes in vascular leakage in the region of the macular in eight patients. Distribution of autofluorescence also showed no absent autofluorescence in the macular area compared to the baseline (Fig. 4c’ and c”), indicating that our injected cells did not lead to further retinal degeneration or oxidative injury to the remaining functional RPE cells. OCT scanning throughout the 24-month follow-up period did not detect the presence of inflammation or cystoid macular edema (Fig. 4 and Additional files 2–7: Figures S2–S7), and none of the patients developed any sign of immunological rejection in the fundus. However, one patient formed a macular membrane (Additional file 1: Figure S1). Given these results, medication for systemic or intraocular immunosuppression was not administered.

**Fig. 5:**
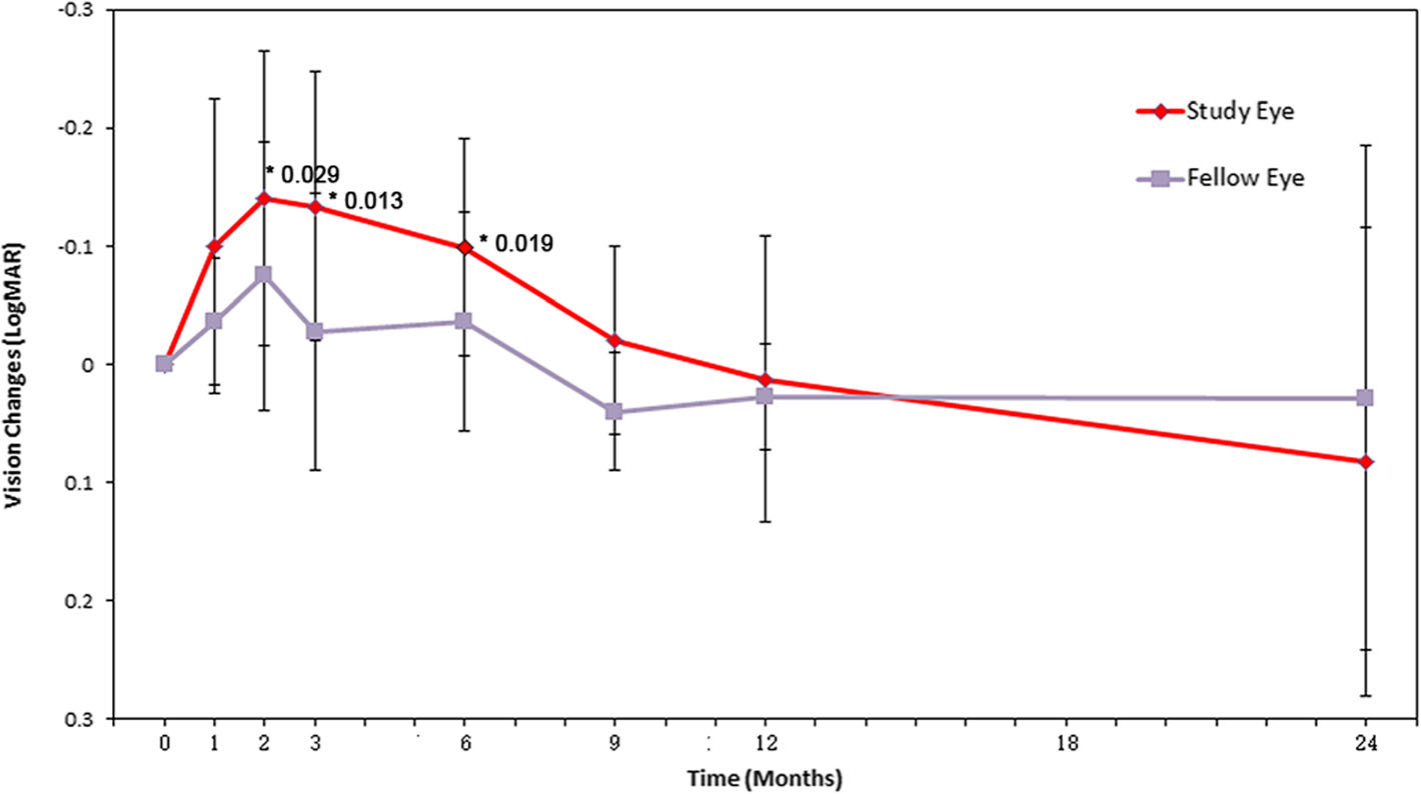
Best corrected visual acuity outcomes following RPC transplantation. The mean best corrected visual acuity (BCVA) in the treated eyes significantly improved, albeit only slightly, at 2, 3, and 6 months compared to the baseline measurements (*P = 0.029, 0.013, and 0.019, respectively; *n* = 8). *MAR* minimum angle of resolution

Visual acuity in the RPC-treated eyes showed a significant improvement for grouped data compared to the baseline (*P* < 0.05) between 2 and 6 months after surgery (Fig. 5, Table 2), but acuity then declined so that overall no differences were seen by 24 months. When individual patients were examined, BCVA improved in five eyes, but remained stable in three eyes during the 12 month follow-up; at 24 months, improved vision was only seen in one eye.

**Table 2:** Visual acuity (logMAR) for patients pre- and post-transplantation

			Follow-up month						
Patient	Eyes	Baseline	1	2	3	6	9	12	24
1	OS	1.56	1.52	1.30	1.30	1.30	1.52	1.80	1.90
	OD	1.58	1.52	1.34	1.52	1.52	1.60	1.60	1.30
2	OS	1.30	1.30	1.20	1.30	1.20	1.30	1.30	1.32
	OD	1.30	1.30	1.30	1.20	1.30	1.30	1.30	1.16
3	OS	1.00	1.00	1.00	0.80	0.80	0.80	0.80	1.00
	OD	0.80	0.80	0.80	0.90	0.90	0.90	0.90	0.90
4	OS	0.60	0.60	0.56	0.54	0.54	0.54	0.56	1.34
	OD	0.34	0.34	0.40	0.42	0.50	0.50	0.54	1.20
5	OS	1.52	1.00	1.00	1.30	1.52	1.60	1.60	1.90
	OD	0.90	0.90	0.90	0.90	0.90	0.90	0.90	1.18
6	OS	1.30	1.00	1.00	1.13	1.26	1.30	1.30	1.15
	OD	0.40	0.30	0.30	0.50	0.40	0.50	0.50	0.70
7	OS	2.00	2.00	2.00	2.00	1.90	2.00	2.00	2.00
	OD	1.90	1.90	1.90	1.90	1.70	2.00	1.90	1.90
8	OS	0.92	0.90	0.90	0.92	0.92	0.92	0.90	
	OD	0.80	0.80	0.80	0.80	0.80	0.80	0.80	

MAR minimum angle of resolution, *OD* right eye without treatment, *OS* left eye with transplant

The recorded signal during global ERG measurements was too small and not clearly distinguishable either before or after RPC transplantation, indicating the poor visual function of the patients prior to the study. Similarly, visual field tests were also unreliable for this reason. Therefore, we developed a computerized pupillometry to assess photoreceptor dysfunction. The thresholds for blue and white light-induced pupillary light reflexes were recorded. The threshold to blue light improved in four subjects (patients 1, 3, 4, and 7) between 3 and 6 months post-transplantation (Additional file 8: Figure S8A, C, D, and G), remained stable in three others (patients 2, 6, and 8; Additional file 8: Figure S8B, F, and H), but declined in one patient (patient 5; Additional file 8: Figure S8E). The threshold to white light improved in patients 1, 3, and 4 (Additional file 8: Figure S8A’, C’, and D’) and remained stable in the other five patients. These findings indicated that, compared to the preoperative baseline, the retina became more photosensitive during the 6-month post-transplantation period in patients 1, 3, and 4, but this was not sustained and threshold levels declined to the baseline at 12 months. In the other five patients, three maintained preoperative baseline levels, while two patients showed a decline in the light reflex.

## Discussion

Clinical trials using hESC-derived RPE cells have been previously attempted [5, 6], and human RPCs derived from the fetus after pregnancy termination are currently being conducted in NIH-approved clinic trials [14, 28]. In contrast to previous clinical trials using fetal retinal transplantation [17–19], our study shows marginal beneficial effects on visual acuity and pupillary responses during the 2- to 6-month follow-up periods, although this efficacy is not maintained in the long term.

Our study was designed to test the safety and tolerability of human RPCs in patients with advanced retinitis pigmentosa; we did not set up cell-dose cohorts due to the small sample sizes. To optimize the chances that the cells would achieve potential efficacy, we selected 1 × 10^6^ RPCs/eye preoperatively in the clinical trial. A previous report suggested that the optimal dose of human RPCs for preserving visual function/retinal structure in dystrophic rats was 0.5 × 10^5^ to 1.0 × 10^5^ cells per injection [28], and for this reason we used ~ 1 × 10^5^ cells per injection in the animal study. This dosage of cells resulted in significant differences between the treated and controlled RCS rats, in agreement with previous authors. Given the size differences between the RCS rat and human retina (80 mm^2^ versus 10 cm^2^) [29], we suggest that an appropriate number of cells needed to gain an improvement of visual acuity would be in the range of ~ 1 × 10^6^ RPCs/eye.

Given the crucial role played by photoreceptors in visual perception, current transplantation strategies aim to replace degenerating photoreceptors as a way of restoring some functional vision. The use of photoreceptor precursors may play a major role in attaining this goal. Unfortunately, a convincing fluorochrome-conjugated antibody that recognizes cell surface antigens is not available and is necessary if photoreceptor precursors are to be efficiently sorted. Singh et al. [30] and Gonzalez-Cordero et al. [31] isolated photoreceptor precursors: respectively a GFP reporter under the control of a neural retina leucine zipper transcription factor or a rhodopsin promoter from the retina in order to sort suitable cells for transplantation. We modified the cell culture method used by Schmitt et al. [14], and confirmed that our human RPCs can partially rescue some visual function in RCS rats. Our results suggest that the improvement in visual response was due to photoreceptor replacement and possibly the secretion of trophic factors from the transplanted cells. In our study, grafted RPCs expressed both recoverin (a photoreceptor and bipolar cell marker) and rhodopsin (a photoreceptor marker). This finding suggests that the transplanted cells are able to differentiate into retinal cells. In addition, eyes with transplants maintained a significantly thicker ONL compared to the control group, which indicates that the preservation of visual function was partially achieved by rescuing the host ONL.

The developmental stage of the donor RPC is important in determining its ability to integrate; early postnatal RPCs integrate into the host ONL with greater efficiency than do late postnatal or adult mature photoreceptors [7, 31]. The RPCs used by Luo et al. [32] were isolated from fetal neural retinas at a gestational age of 16 weeks; however, little evidence of replacement of degenerated photoreceptors with donor cells was confirmed [32]. Their study indicated that trophic factors played a major role in rescuing endogenous photoreceptors via RPCs, with a greater number of preserved ONL cells. Our RPCs were collected from slightly younger fetuses (12–16 weeks). These RPCs might be better committed to cell fate compared to those used by Luo et al. [32], and therefore are likely to follow a more appropriate differentiation which would account for differences in our results compared to the data of Luo et al.

Although our animal study demonstrated that human RPC transplantation preserved the vision of RCS rats, a similar statistical improvement in vision between 6 to 24 months was not seen in the clinical trial. A major difference between our human trial and animal studies is that the human recipients were generally at an advanced stage of retinal degeneration compared to the earlier stage of the disease process in the RCS rats. Chronic inflammatory reaction is present in the eyes of patients with retinitis pigmentosa and plays an important role in the pathogenesis of retinitis pigmentosa [33]. As the retinal degeneration aggravates, more microglial cells in the dystrophic retina are activated gradually, and inflammatory factors secreted by microglial cells will increase accordingly, which is actually detrimental to donor cell survival [34]. The subretinal space of the host retina in the earlier disease process of the rats may provide a more suitable environment for donor cells, resulting in better survival and functional improvement. In addition, cortical changes occur following visual loss, including retinitis pigmentosa, that also seriously affect visual perception of phoshene [35, 36]. These results indicate that the baseline status of a recipient’s retinal function may have a direct impact on postoperative recovery and should be taken into consideration in devising future transplantation strategies.

It was difficult to compare the pupillary diameter changes during the visual function analysis because the pupillary light reflex (PLR) tests were variable and each test lasted for over 40 min. However, the threshold in our study was stable in three repeated tests. Therefore, we adopted pupillary threshold changes to objectively represent retinal function. We did not find an association between improvements in the PLR and visual acuity for each subject; however, the trend was identical in showing improved visual function from 3 to 6 months post-transplantation. Differences between visual acuity and the PLR test no doubt arise because visual acuity mainly reflects foveal function, particularly cone sensitivity. During the PLR test, we most likely activated primarily rods under scotopic conditions by using a dimmer blue or white light stimulus [37].

In our study, clinical examination observed that allogeneic transplantation of human fetal-derived RPCs into the diseased retina did not induce any signs of apparent rejection, such as local retinal inflammation, vascular leakage, or neovascularization. It is known that the subretinal space possesses relative immune privilege [38, 39] and animal experiments have shown that fetal neural retinas have low immunogenicity [21]. Immunological rejection was not observed after transplantation of retinal cells or retinal tissue together with retinal pigment epithelium from human fetuses into the subretinal space of retinitis pigmentosa patients [19, 40]. In agreement with previous studies, we did not observe any signs of immunological rejection in response to RPC transplants, confirming this strategy is a safe procedure.

## Conclusions

Our major finding is that fetal RPCs can be safely transplanted into the retinas of retinitis pigmentosa patients. These results provide useful information for future investigations related to cell-based therapies for the treatment of retinitis pigmentosa and other inherited retinal degenerations.

## Additional files

Additional file 1: Figure S1. Morphologic changes after RPC transplantation into the retina of patient 1. Color fundus photographs, fluorescein angiograms (FA), and OCT images are shown pre- and postoperatively. OCT showed macular membrane formation at the 12-month follow-up. OCT ocular coherence tomography. (TIF 2469 kb)

Additional file 2: Figure S2. Morphologic changes in patient 2. (TIF 2215 kb)

Additional file 3: Figure S3 Morphologic changes in patient 3. (TIF 1489 kb)

Additional file 4: Figure S4 Morphologic changes in patient 4. (TIF 2045 kb)

Additional file 5: Figure S5. Morphologic changes in patient 5. (TIF 1318 kb)

Additional file 6: Figure S6. Morphologic changes in patient 7. (TIF 2092 kb)

Additional file 7: Figure S7. Morphologic changes in patient 8. (TIF 2646 kb)

Additional file 8: Figure S8. Pupil responses in all patients after RPC transplantation. (A–H) Figures show pupillary light reflex (PLR) elicited by blue stimuli, while (A’–H’) show PLR elicited by the white stimuli. (A, C, D, G) thresholds decreased after 3 to 6 months indicating patients were more photosensitive, but by 12 months thresholds had returned to baseline. Using a white stimulus (A’, C’, D’, E’) produces similar results. (TIF 1077 kb)

Additional file 9: Video S1. Injection of transplanted cells. After vitrectomy, a 39G cannula was used to create a bleb space for the injection of transplanted cells. (WMV 29837 kb)

## Abbreviations

BCVA: Best corrected visual acuity
bFGF: Basic fibroblast growth factor
CRX: Cone-rod homeobox-containing gene
EGF: Epithelial growth factor
ERG: Electroretinography
ESC: Embryonic stem cell
FA: Fluorescein angiography
FACS: Fluorescence-activated cell sorting
fERG: Flash electroretinography
GFAP: Glial fibrillary acidic protein
GMP: Good Manufacturing Practice
hESC: Human embryonic stem cell
iPSC: Induced pluripotent stem cell
ISCEV: International Society for Clinical Electrophysiology of Vision
MAR: Minimum angle of resolution
OCT: Optical coherence tomography
ONL: Outer nuclear layer
PAX6: Paired box protein 6
PBS: Phosphate-buffered saline
PLR: Pupillary light reflexes
RCS: Royal College of Surgeons
RPC: Retinal progenitor cell
RPE: Retinal pigment epithelial
RT-qPCR: Real-time quantitative polymerase chain reaction
Sox2: SRY (sex determining region Y)-box 2
