# Supplementary figures and images for: Long-term safety of human retinal progenitor cell transplantation in retinitis pigmentosa patients
Source: Stem Cell Res Ther. 2017 Sep 29;8:209. doi: 10.1186/s13287-017-0661-8 (PMC5622579; doi:10.1186/s13287-017-0661-8)

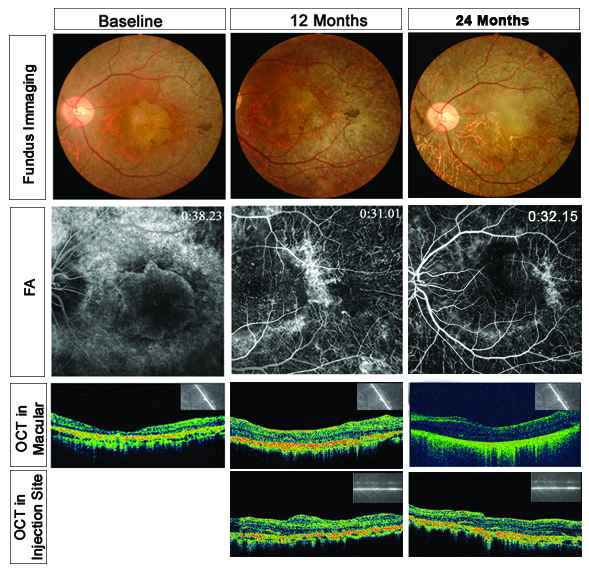

Supplement: Supplementary file 1 — Morphologic changes after RPC transplantation into the retina of patient 1. Color fundus photographs, fluorescein angiograms (FA), and OCT images are shown pre- and postoperatively. OCT showed macular membrane formation at the 12-month follow-up. OCT ocular coherence tomography. (TIF 2469 kb) [file 13287_2017_661_MOESM1_ESM.tif]

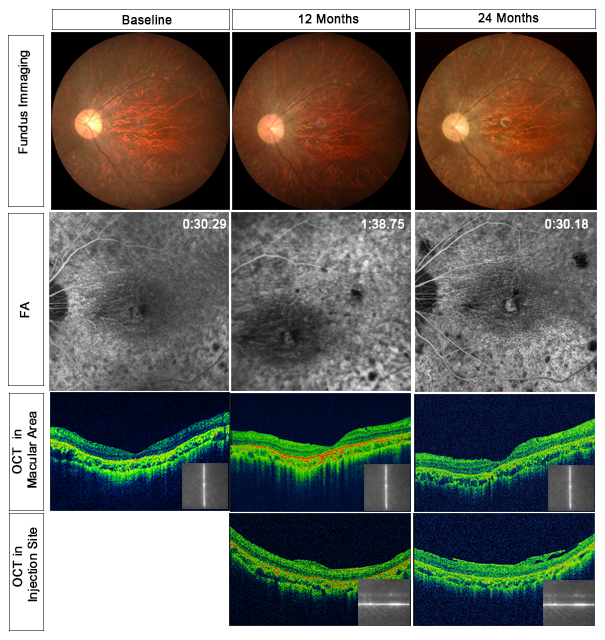

Supplement: Supplementary file 2 — Morphologic changes in patient 2. (TIF 2215 kb) [file 13287_2017_661_MOESM2_ESM.tif]

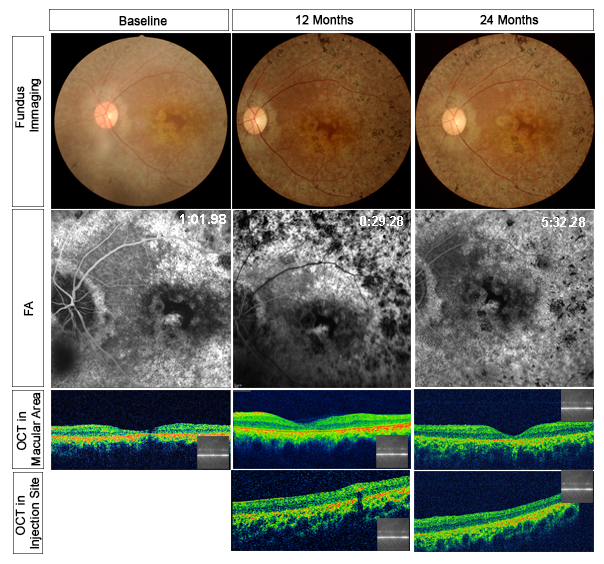

Supplement: Supplementary file 3 — Morphologic changes in patient 3. (TIF 1489 kb) [file 13287_2017_661_MOESM3_ESM.tif]

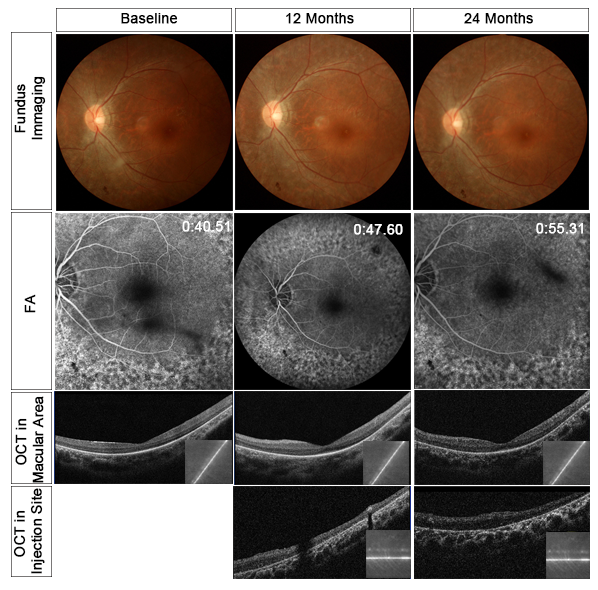

Supplement: Supplementary file 4 — Morphologic changes in patient 4. (TIF 2045 kb) [file 13287_2017_661_MOESM4_ESM.tif]

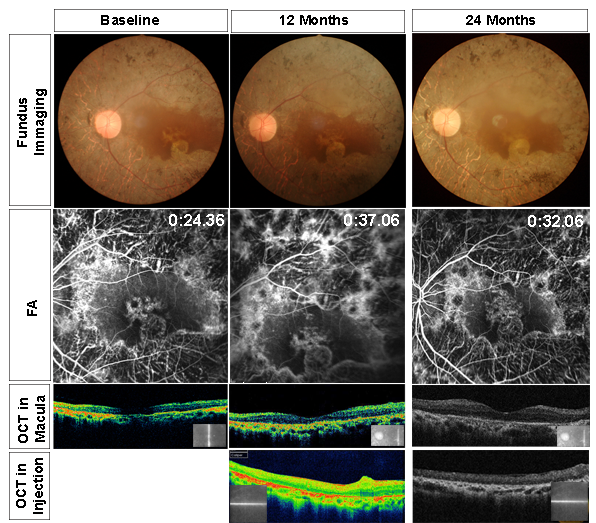

Supplement: Supplementary file 5 — Morphologic changes in patient 5. (TIF 1318 kb) [file 13287_2017_661_MOESM5_ESM.tif]

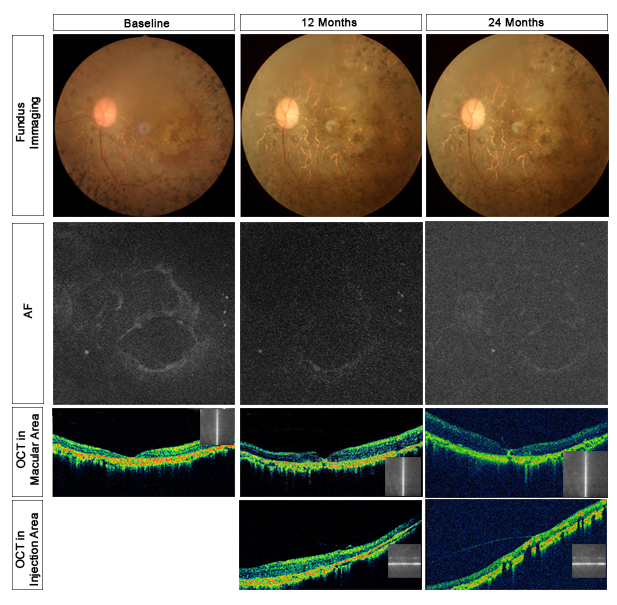

Supplement: Supplementary file 6 — Morphologic changes in patient 7. (TIF 2092 kb) [file 13287_2017_661_MOESM6_ESM.tif]

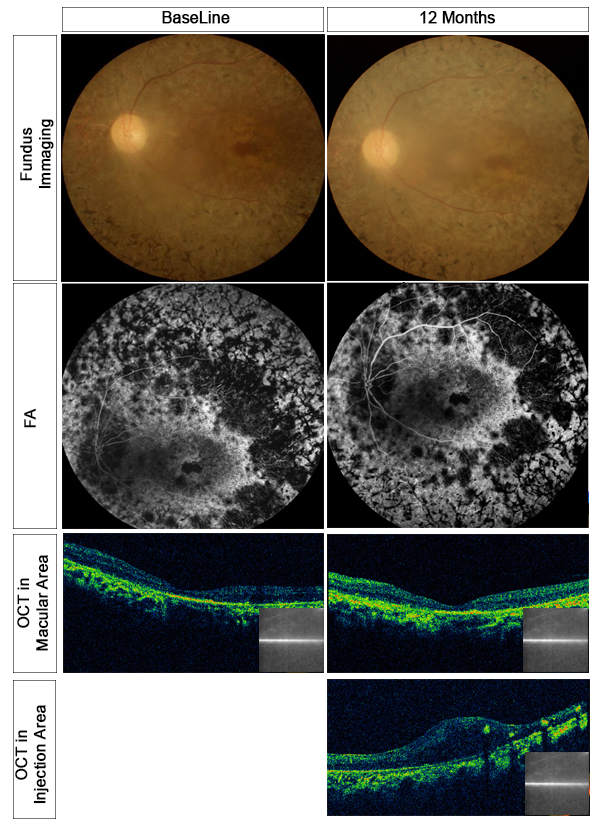

Supplement: Supplementary file 7 — Morphologic changes in patient 8. (TIF 2646 kb) [file 13287_2017_661_MOESM7_ESM.tif]

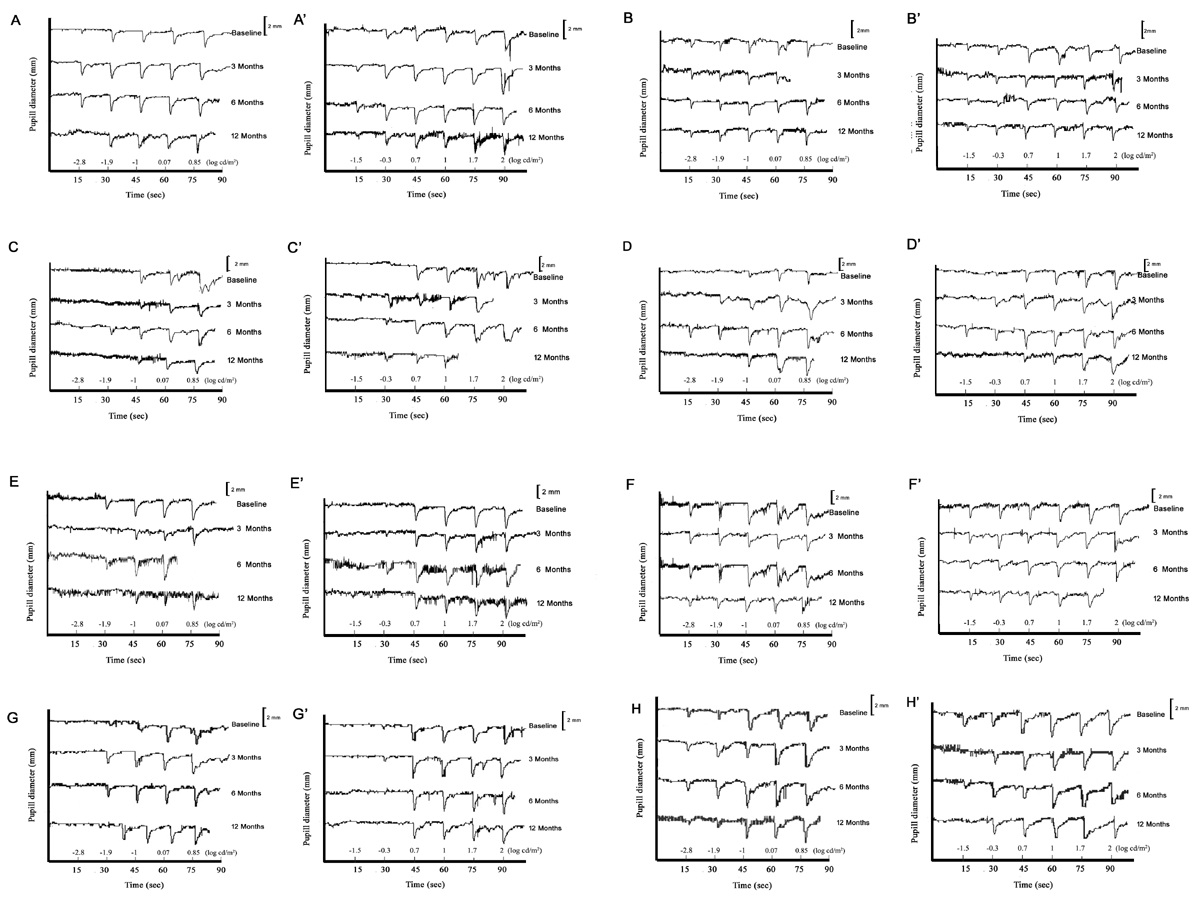

Supplement: Supplementary file 8 — Pupil responses in all patients after RPC transplantation. (A–H) Figures show pupillary light reflex (PLR) elicited by blue stimuli, while (A’–H’) show PLR elicited by the white stimuli. (A, C, D, G) thresholds decreased after 3 to 6 months indicating patients were more photosensitive, but by 12 months thresholds had returned to baseline. Using a white stimulus (A’, C’, D’, E’) produces similar results. (TIF 1077 kb) [file 13287_2017_661_MOESM8_ESM.tif]
